# A Cytokine Network Balance Influences the Fate of *Leishmania (Viannia) braziliensis* Infection in a Cutaneous Leishmaniasis Hamster Model

**DOI:** 10.3389/fimmu.2021.656919

**Published:** 2021-07-01

**Authors:** Milla B. Paiva, Raquel Peralva Ribeiro-Romão, Larissa Resende-Vieira, Thais Braga-Gomes, Marcia P. Oliveira, Andrea F. Saavedra, Luzinei Silva-Couto, Hermano G. Albuquerque, Otacilio C. Moreira, Eduardo Fonseca Pinto, Alda Maria Da-Cruz, Adriano Gomes-Silva

**Affiliations:** ^1^ Laboratório Interdisciplinar de Pesquisas Médicas, Instituto Oswaldo Cruz, FIOCRUZ, Rio de Janeiro, Brazil; ^2^ Laboratório de Transmissores de Hematozoários, Instituto Oswaldo Cruz, FIOCRUZ, Rio de Janeiro, Brazil; ^3^ Laboratório de Biologia Molecular e Doenças Endêmicas, Instituto Oswaldo Cruz, FIOCRUZ, Rio de Janeiro, Brazil; ^4^ Rede de Pesquisas em Saúde do Estado do Rio de Janeiro/FAPERJ, Rio de Janeiro, Brazil; ^5^ Disciplina de Parasitologia-DMIP, Faculdade de Ciências Médicas, UERJ, Rio de Janeiro, Brazil; ^6^ The National Institute of Science and Technology on Neuroimmunomodulation (INCT-NIM), Rio de Janeiro, Brazil; ^7^ Laboratório de Pesquisa Clínica em Micobacterioses, Instituto Nacional de Infectologia Evandro Chagas, FIOCRUZ, Rio de Janeiro, Brazil

**Keywords:** cytokines, cutaneous lesions, parasite load, disease control, *Leishmania (Viannia) braziliensis*, iNOS/arginase, immune-regulation, hamster model

## Abstract

The golden hamster is a suitable model for studying cutaneous leishmaniasis (CL) due to *Leishmania *(*Viannia*) *braziliensis.* Immunopathological mechanisms are well established in the *L. (L.) major*-mouse model, in which IL-4 instructs a Th2 response towards progressive infection. In the present study, we evaluated the natural history of *L.* *braziliensis* infection from its first stages up to lesion establishment, with the aim of identifying immunological parameters associated with the disease outcome and parasitism fate. To this end, hamsters infected with 10^4^, 10^5^, or 10^6^ promastigotes were monitored during the first hours (4h, 24h), early (15 days, 30 days) and late (50 days) post-infection (pi) phases. Cytokines, iNOS and arginase gene expression were quantified in the established lesions by reverse transcription-quantitative PCR. Compared to the 10^5^ or 10^6^ groups, 10^4^ animals presented lower lesions sizes, less tissue damage, and lower IgG levels. Basal gene expression in normal skin was high for TGF-β, and intermediary for TNF, IL-6, and IL-4. At 4hpi, no cytokine induction was observed in the 10^4^ group, while an upregulation of IL-6, IL-10, and IL-4 was observed in the 10^6^ group. At 15dpi, lesion appearance was accompanied by an increased expression of all assessed cytokines, markedly in the 10^5^ and 10^6^ groups. Upregulation of all investigated cytokines was observed in the late phase, although less expressive in the 10^4^ group. IFN-γ was the depending variable influencing tissue damage, while IL-6 was associated to parasite load. The network correlating gene expression and clinical and laboratorial parameters indicated inoculum-independent associations at 15 and 30dpi. A strong positive network correlation was observed in the 10^4^ group, but not in the 10^5^ or 10^6^ groups. In conclusion, IL-4, IL-6, IL-10, and TGF-β are linked o *L. braziliensis* progression. However, a balanced cytokine network is the key for an immune response able to reduce the ongoing infection and reduce pathological damage.

## Introduction

Tegumentary leishmaniasis (TL) is an infectious disease associated with poverty, and a general increasing trend in the number of new cases isreported annually to World Health Organization (1). The disease is caused by a protozoan belonging to the *Leishmania* genus, which compromises the skin and mucosa. Cutaneous leishmaniasis (CL) is the most common clinical form, characterized by one or more skin ulcers well delimited by a raised border at the site of the sand-fly infected bite. The *Leishmania* species implicated in CL can vary according to geographic region, and eight species have been identified in Brazil (2, 3). Although most patients develop cutaneous ulcers, increasing evidence indicates clinical presentation characteristics associated with a particular *Leishmania* species (4–7). Because of this, the understanding of CL physiopathology must be particular to each *Leishmania* species. *L. (Viannia) braziliensis* is the most spread parasite in Brazil, causing not only CL but also mucosal leishmaniasis (ML), a more severe clinical form of the disease. Clinical aspects variability indicate that, besides the genetic host background, antigenic strain differences of this *Leishmania* species also drive infection fates (8–10).

*L. braziliensis* promastigotes migrate to draining lymph nodes after the insect bite, leading to the following possible outcomes: 1) infection control and subject evolution to either oligo or asymptomatic (11, 12); 2) disease progress to nodules (early-CL), which increase in size and ulcerate (late-CL) (13); 3) clinical lesion cure after anti-leishmanial therapy or spontaneous healing (7, 14, 15); 4) evolution to mucosal leishmaniasis in 3 to 5% of infected subjects (16). Pioneer studies applying experimental animal models (17–19) and, to a lesser amount, in humans (20–23) have shown that the early infection phase is crucial in determining *Leishmania* infection fate. Infective burden (24), cytokine balance (25), infection site (26) and infection-site infiltrating leukocyte dynamics (27, 28) during the very early infection stage seem to dictate and determine the evolution of the disease.

During the natural history of this infection, *L. (L.) major*-C57BL/6 model parasites slowly multiply during the first 2 weeks, reaching a peak around the 4^th^ week post-infection (pi). This “silent phase” coincides with increases in IL-4 expression. IFN-γ increased from the 4^th^ week, coinciding with the appearance of nodular lesions (5^th^ week) and decreasing parasite loads (19). In BALB/c mice, rapid IL-4 production soon after *L. major* infection is necessary and sufficient to instruct Th2 cell development, resulting in disease progression (18, 25, 29, 30). However, little attention is given to the mechanisms underlying the first steps of the parasite-host relationship that influence different *L. braziliensis* infection fates.

We have previously demonstrated that hamsters infected with 10^4^ *L. braziliensis* promastigotes develop smaller lesions exhibiting less severe clinical aspects and tissue inflammatory infiltrate compared to animals infected by 10^5^or 10^6^ inocula. Despite this, high IFN-γ gene expression, anti-*Leishmania* IgG levels, and parasite load occurred independently of inocula size. However, nodule appearance onset is indirectly correlated to the number of inoculated parasites, indicating that parasite loads influence the clinical course of leishmaniasis (31). This prompted the investigation of early immunological profiles associated to favorable prognoses of infected animals exposed to lower parasite loads (10^4^) in comparison to those developing the severe form of disease, challenged with higher parasite inocula (10^5^ or 10^6^). Therefore, a study was designed in which an *L. braziliensis*-hamster outbred model challenged with three different parasite burden tracked at the main disease stages would expect to mirror the natural history of CL in humans.

## Material and Methods

### Animals and Ethics Statements

Adult female outbred golden hamsters (*M. auratus*) (6-10 weeks old), weighing 80-90 g, obtained from the animal facilities belonging to the Fundação Oswaldo Cruz (FIOCRUZ), were used. Ninety-six (96) infected animals (four/group) were included: a) one experiment for 4- and 24 hpi; b) two experiments for 15-, 30- and 50 days post infection. Sixteen uninfected animals were used as control. This study was performed in two independent experiments and approved by the Ethics Committee on Animal Use (CEUA) of FIOCRUZ, with protocol number IOC 032/15.

### Parasites, Infection and Experimental Protocol

*L. (V.) braziliensis* (MCAN/BR/98/R619) in the stationary growth phase from the third *in vitro* passage were resuspended in a total volume of 20 µL of phosphate-buffered saline (PBS). Inocula containing 1×10^4^, 1×10^5^ or 1×10^6^ parasites were used for intradermal inoculation into the dorsal hind paw of hamsters, as described (31). Animals were euthanized 4- and 24-hours post-infection (hpi) and 15-, 30- and 50-days dpi through administration of the preanesthetic medications Ketamine and Xylazine (200 and 10mg/kg, respectively) injected intraperitoneally and, after sedation, sodiumthiopental at a dose of 150 mg/kg, also intraperitoneally. The death of the animals was confirmed by cardiac arrest.

### Clinical Evaluation of *Leishmania (Viannia) Braziliensis* Infection

**Figure 1:**
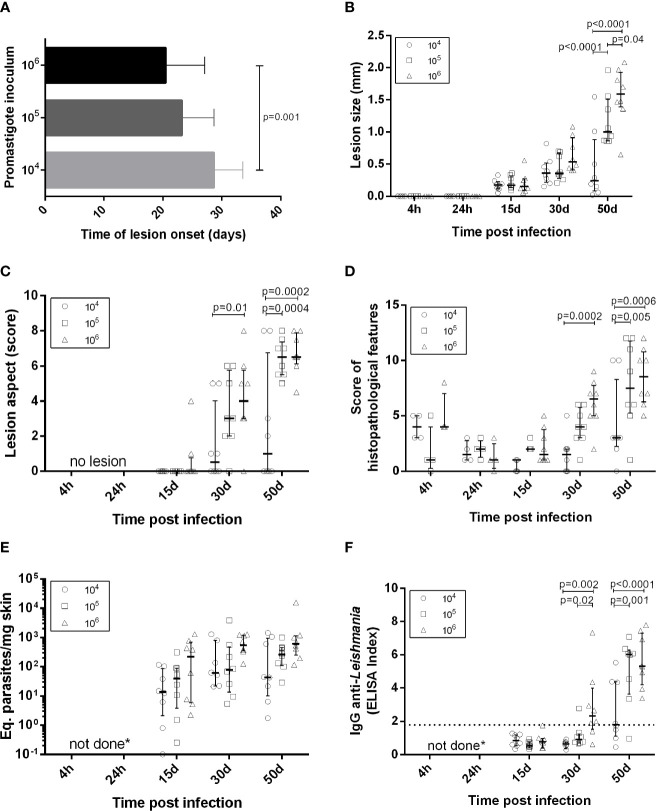
Clinical, parasitological and immunological aspects in golden hamsters infected with *Leishmania (Viannia) braziliensis*. The animals were followed-up during the first hours (4h, 24h), early (15 days and 30 days) and late (50 days) post-infection phases. **(A)** Time of lesion onset; **(B)** Lesion size was defined by the difference in millimeters (mm) between the thickness of the infected and the non-infected paw of the same animal; **(C)** Lesions scores (s) were defined as follows: no lesion (s=0), edema (s=1), discrete papule (s=2), moderate papule (s=3), discrete nodule (s=4), moderate nodule (s=5), large nodule (s=6), very large nodule (s=7), and, if ulcers were present, associated with early ulcer (s=0.5), ulcer (s=1), ulcer with crust (s=1.5), deep ulcer (s=2) **(D)** Regarding histopathological scores: no observation (score=0), slight (score=1), moderate (score=2) and intense (score=3); **(E)** Parasite loads were quantified in the lesions by qPCR and the results were expressed as Equivalent parasites per milligram of skin; **(F)** Anti-IgG levels were quantified in plasma by an ELISA and the results were expressed as an Elisa Index. 10^4^ (o), 10^5^ (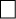), 10^6^(**Δ**) –number of promastigotes used for infection; Each symbol represents one animal; The lines in each scatter group represent the median and interquartile range; the dotted line indicates the cutoff value for IgG quantitation; *p* – Statistical significance.

The skin lesions were monitored weekly from day 7 up to 50 dpi, measuring the paw dorsum-ventral thickness with a digital thickness gauge (Mitutoyo America Corporation, São Paulo, Brazil). The lesion size was determined by the difference in millimeters between the thickness of the infected and the non-infected paw of the same animal. Clinical aspects of the lesions were qualified with a score system (**Figure 1C**).

### Quantification of Anti-*Leishmania* Antibodies

The levels of IgG anti-*Leishmania* were determined by an ELISA assay in house (Enzyme-linked immunosorbent assay) as previously described with some adaptations (32). Briefly, *L. braziliensis* (MHOM/BR/1975/M2903) soluble antigen (2 µg/well) were adsorbed in microtiter plate (Nunc-immuno Plate, Roskilde, Denmark). Then, hamsters’ plasma samples were diluted 1:50 and added in triplicate. Horseradish peroxidase labelled goat anti-hamster IgG was used as detector system (Santa Cruz Biotechnology, Santa Cruz, CA, USA). The results were expressed as ELISA index (EI), obtained by mean of sample absorbance divided by mean of negative controls (n=5) absorbance. The cut off was determined by ROC method (Receiver Operator Characteristic Curve).

### Histopathological Analyses

Fragments from the skin of the infected paw were fixed in 4% paraformaldehyde solution and processed for paraffin embedding. Sections of 5 μM thickness were stained with haematoxylin-eosin and then observed by light microscopy (Nikon Eclipse E600, Microscope, Tokyo, Japan). Results from the skin histopathological analysis were expressed by a score criteria (31), based on a semi-quantitative analysis that evaluated the intensity of each histopathological features occurred: presence of inflammatory infiltrated, bleeding, congestion of vessels, granulomas, vacuolated macrophages, *Leishmania* amastigotes, Schaumann’s bodies and/or necrosis (**Figure 1D**).

### Parasites Detection by Immunohistochemistry

Another skin fragment was embedded in OCT compound (ornithine carbamyltransferase compound; Tissue Tek, Illinois, USA). Frozen tissue sections (4 μM) were cut at −27°C and mounted on poly-L-lysine (Sigma Chemical Co., Saint Louis, USA) coated slides. The slides were dried, fixed in cold acetone (Merck, Darmstadt, DE) for 10 minutes and permeabilized with 0.4% Triton X-100 (Sigma, USA) in Tris-HCl. Then, samples were incubated with 0.6% hydrogen peroxide to block endogenous peroxidases and were then incubated with 2% bovine serum albumin (Sigma, USA) to inhibit non-specific binding. Heterologous serum (primary antibody) collected from mice infected with *L. (L.) infantum* (IFLA/BR/1967/PH8) was incubated overnight at 4°C in humidified chamber diluted 1:100. The Polymer Detection System for Human Tissue (Abcam, Cambridge, UK) was used for immunohistochemical staining following the manufacturer’s instructions. The reaction was revealed using aminoethylcarbazole as chromogen. Nuclei were counterstained with Mayer’s hematoxylin (Merck), and the slides were mounted in aqueous mounting medium. Normal mouse or goat serum was used as negative control.

### Cytokine, Arginase and iNOS Gene Expression by Reverse Transcription Quantitative PCR (RT-qPCR)

Cytokines and enzymes gene expression were quantitated from skin lesion fragment using a combination of TRIzol^®^ (Invitrogen, California, USA) and RNeasy^®^ minikit (Qiagen, Austin, Texas, USA) for RNA extraction (water phase). The evaluation of IFN-γ, TNF, IL-6, IL-10, TGF-β, IL-4, iNOS, arginase and housekeeping GAPDH and γActin mRNA were based on the protocol previously described (33). Relative quantitation of gene expression was calculated using the comparative cycle threshold (Ct) method (ΔΔCt), as previously described (34), with threshold set at 0.02. Gene expression was represented as fold change (2-ΔΔCt), in relation to skin samples from uninfected hamsters, used as calibrators. The Expression Suite Software (Thermo Fisher Scientific, Massachusetts, USA) was used for analysis.

### Parasite Load Detection by Quantitative PCR (qPCR)

Parasite load was quantified from the same skin lesion fragment used to determine mRNA expression, using the TRIzol^®^ protocol for DNA extraction (organic phase). The measurement of parasite load by qPCR was performed by absolute quantitation, based on a standard curve produced from DNA samples extracted from fragments of hamster skin, artificially infected with promastigote forms of *L. braziliensis*. For parasite quantitation, primers targeting the conserved regions of kinetoplastid DNA minicircles (kDNA) were used (35). The parasite load was calculated by the (*L. braziliensis* equivalents/hamster skin mass equivalents) ratio and expressed as “parasite load” (parasites eq/mg skin).

### Statistical Analyses

Comparison between experimental groups was performed using one-way analysis of variance (ANOVA). Statistical tests from the ΔCt values were student t-test or Mann-Whitney rank sum test, and Analysis of Variance. Spearman’s correlation matrix was performed using ΔCt values for gene expression. These analysis were performed with GraphPad Prism software version 6.0 for Windows (GraphPadSoftware, San Diego, CA, USA) or SigmaPlot v12.0 software (Systat Software, Inc). A multivariate statistical analysis was performed through multiple linear regression (SPSS software, version 9.0) to determine the influence of intervening variables. Heatmap matrix analyses were performed for gene expression ΔΔCt using online software Heat mapper^®^ (Wishart Research Group at the University of Alberta). The hierarchical clustering method used for analysis was the average linkage, and the distance measurement method applied was Euclidean. The interaction network was done with correlations that presented significance level ≤0.05 using Cytoscape 3.7.2 software. The clinical and laboratorial results were expressed as medians with interquartile ranges. All RT-qPCR and qPCR assays were performed in experimental duplicates and results were expressed as median ± standard deviations. Significant differences were considered when *p* values were ≤0.05.

## Results

### Low *Leishmania (Viannia) braziliensis* Infection Is Associated With a Better Clinical Prognosis

Lesion development kinetics (**Figure 1B**), confirmed a progressive course of evolution towards the chronic disease phase. An inverse relationship between parasitic inoculum concentration and lesion time onset was reproduced (**Figure 1A**) (31). At 50 dpi, the median lesion size in animals infected with 10^5^ parasites was significantly higher (1.00 mm [0.86 – 1.51mm]) when compared to the 10^4^ group (0.24 mm [0.08 – 0.88 mm]), but lower than the 10^6^ group (1.59 mm [1.39 – 1.93 mm]).

A variable lesion increment pattern was observed, especially at 50 dpi. At this time point, most animals infected with 10^4^ parasites (six out of eight) presented less than 0.5 mm swelling. Two animals, however, exhibited lesions as large as those observed in the 10^5^ or 10^6^ groups. On the other hand, one out of eight animals infected with 10^6^ parasites developed a smaller lesion (0.65 mm) than the median observed in this group (1.6 mm) (**Figure 1B**). Most animals in the10^4^ group presented less severe lesions than those infected with 10^5^ and 10^6^ parasites (**Figure 1C**). Curiously, despite differences in lesion size, these two groups presented similar clinical sign scores and the predominance of nodular and ulcerated lesions, considered a more destructive outcome. Similar patterns were observed concerning the histopathological scores (**Figure 1D**).

### Anti-*Leishmania* IgG Is Higher in Animals Infected With 10^5^ and 10^6^ Promastigote Loads Compared to 10^4^

Results were expressed as the ELISA index (**Figure 1F**). No specific IgG production was observed in any of the three experimental groups at 15 dpi. At 30 dpi, most animals from the 10^4^ and 10^5^ groups maintained IgG anti-*Leishmania* levels under the cut-off (except a single 10^5^ infected animal), while most infected by 10^6^ parasites already exhibited seroconverted IgG levels. At 50 dpi, IgG levels in the10^4^ group were significantly lower than those in the 10^5^ and 10^6^. Animals presenting IgG under the cut-off at 50 dpi were similar, exhibiting lesion size less than 1.0 mm (**Figure 1B**) and parasite load under 10^2^ (**Figure 1E**).

### Arginase and iNOS Expression Do Not Determine Parasite Loads in Hamsters Infected With *Leishmania (Viannia) braziliensis*

**Figure 2:**
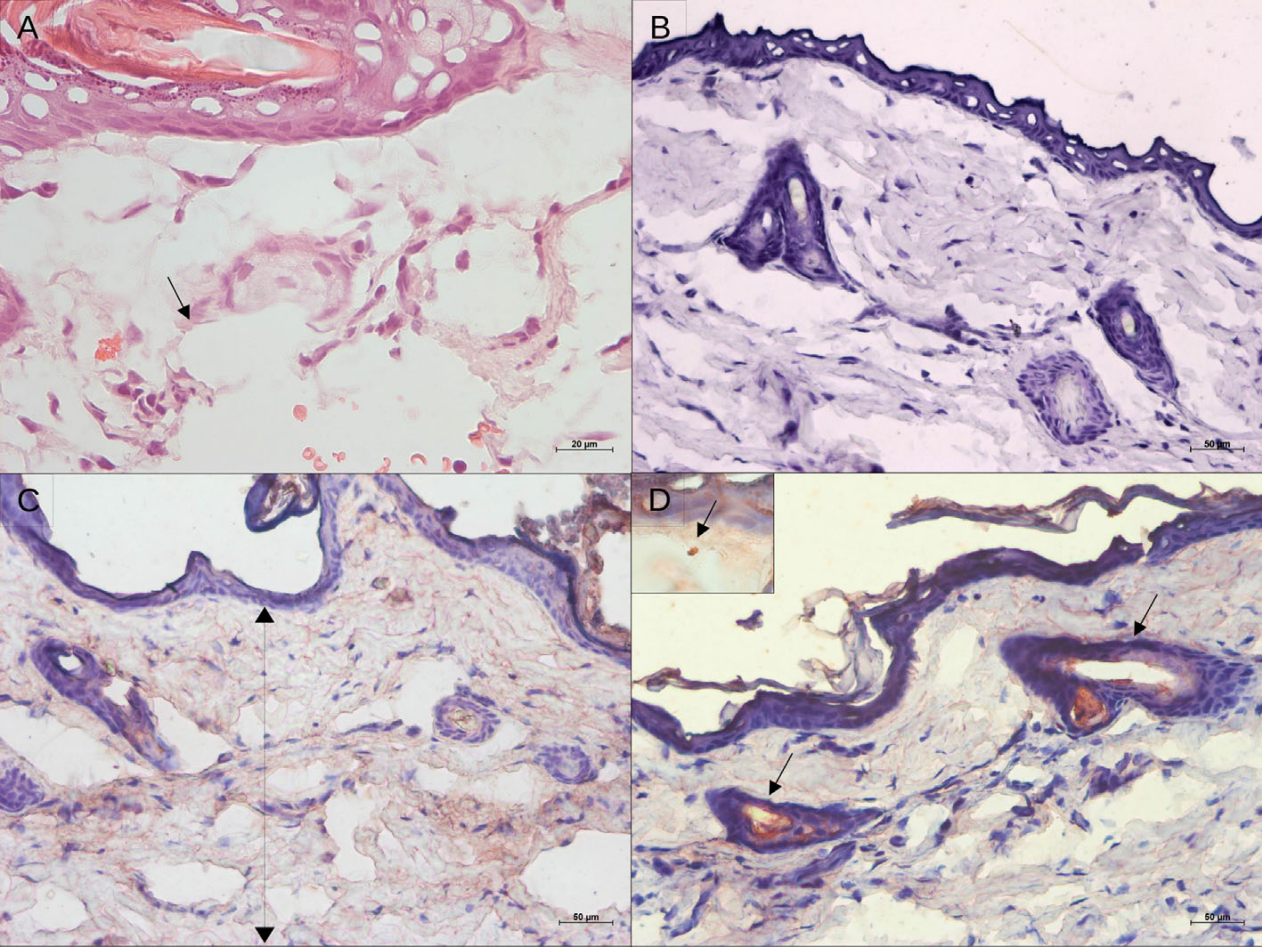
Histological aspect and leishmanial antigen detection in the skin of golden hamsters during early after *Leishmania *(*Viannia*) *braziliensis* infection. **(A)** Epidermal and dermal structure at 4 hours pos-infection, showing no tissue disturbance, only intumescent lymphatic vessels (hematoxylin and eosin; 20X magnification); **(B)** Immunohistochemistry control; **(C)** Immunostaining for *Leishmania* antigens showing antigens spread throughout the tissue at 4h, and **(D)** Detection of leishmanial antigens at 24 hpi showing concentrated staining of the nearest pilous follicle (50X magnification). The insert displays an amastigote.

DNA parasite quantitation in lesions at 15 dpi was similar in all groups (**Figure 1E**). At 50 dpi, it is noteworthy that animals from the 10^4^ group presented variable amounts of parasite loads. Instead, the 10^5^ and 10^6^ groups exhibited similar pattern sand lower variability in comparison to the 10^4^ group. Surprisingly, no parasites were observed in the histopathological analysis at 4 hpi. Immunohistochemistry assessments indicated diffuse staining throughout the dermis, but no integral parasites. At 24 hpi, staining was more concentrated in sebaceous glands and hair follicles and amastigote groups were identified (**Figure 2**). No inflammatory cells were observed.

**Figure 3:**
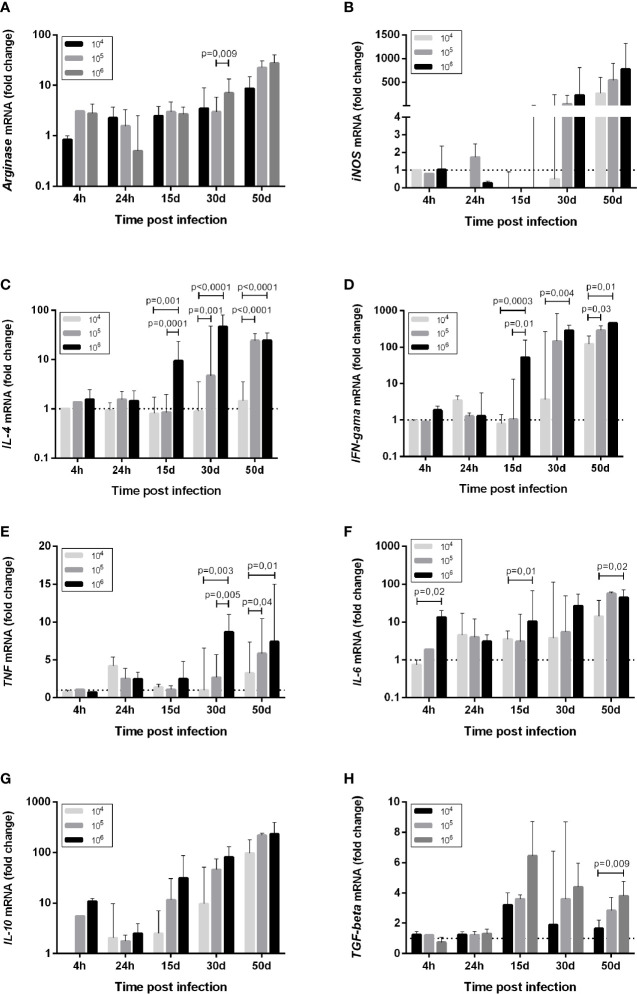
Cytokine mRNA expression **(A)** *Arginase*, **(B)** *iNOS*, **(C)** *IL-4*, **(D)** *IFN-gama*, **(E)** *TNF*, **(F)** *IL-6*, **(G)** *IL-10*, **(H)** *TGF-beta*, in the skin of golden hamsters at different time points after infection with different *Leishmania* (*Viannia*) *braziliensis* inocula. The animals were infected with three parasite concentrations (10^4^, 10^5^, 10^6^ promastigotes). Skin mRNA expression was determined by RT-qPCR at different times post infection, namely the first hours (4h, 24h), early (15 days and 30 days) and late (50 days) post-infection phases. The dotted line represents the basal expression of non-infected skin. The results are expressed as relative fold changes between the experimental samples and the skin of an uninfected animal, to which a p – statistical significance value 1 was arbitrarily assigned. The bar represents the median and the line, the interquartile ranges. 10^4^, 10^5^, 10^6^ - number of promastigotes used for infection.

**Figure 4:**
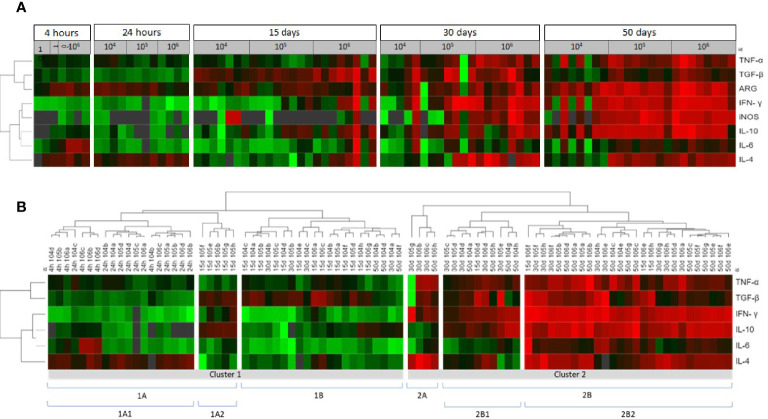
Cytokine, iNOS and arginase in the skin lesions of hamsters infected with *Leishmania (Viannia) braziliensis* according to a comparative gene expression heatmap analysis. **(A)** The animals were grouped by post infection time (4 and 24 hours; and 15, 30 and 50 days) and different inocula (10^4^, 10^5^, and 10^6^ promastigotes). Heatmap analyses were performed for gene expression (*tnf, tgfβ, ifnγ, il10, il6, il4, arg, inos*) ΔΔCt using the online Heat mapper^®^ software. **(B)** The animals were clustered by gene expression signature. The hierarchy average linkage clustering method with Euclidean distance measurement was used (Wishart Research Group at the University of Alberta). Each row represents one molecule and each column represents one animal. Higher gene expression is displayed in red, and lower gene expression, in green. Gray indicates mean gene expression below the lower limit of detection.

Arginase, an enzyme found in all *Leishmania* strains and produced by macrophages, stimulates parasite replication and inhibits nitric oxide function (36). The basal *arg* expression was high in hamster skin. At 4 hpi, *arg* expression was upregulated compared to normal skinin the 10^5^ and 10^6^groups, but not in the 10^4^ group. After 24 hpi, an *arg* increase was also observed in the 10^4^ group. All three inocula groups exhibited around a 5-fold increase in *arg* expression at 15 and 30 dpi, and around a 10-fold increase at 50 dpi (**Figures 3A**, **4A**). This environment is accompanied by elevated parasite loads (**Figure 1E**).

Macrophage microbicidal function was evaluated by iNOS gene expression (**Figures 3B**, **4A**). Basal iNOS expression in hamster skin is low and was down-modulated or even absent (gray color) during the first hours (4 and 24 hpi), as well as at 15 dpi, independent of the inocula. At 30 dpi, a similar trend was observed for animals from the 10^4^ group and in half of the animals infected with 10^5^, while the 10^6^ group exhibited a high expression of this enzyme. At 50 dpi, high iNOS expressions were detected in half of the eight animals from the 10^4^ group and in all the animals from 10^5^ and 10^6^ groups. However, this iNOs expression increase was not associated with parasite load control (37).

### Promastigote Infective Loads Dictate the Parasite-Induced Cytokine Pattern

Changes in the cytokine gene expression of infected skin compared to uninfected skin were assessed. At the moment of infection, the promastigotes encountered an environment of high TGF-β gene expression and intermediary basal TNF, IL-6, and IL-4 gene expressions. Concerning the 10^4^ group, promastigotes did not trigger an increase in IL-4 expression at 4 hpi (**Figure 3**). Basal IL-4 levels were maintained up to 30 dpi, and a discrete upregulation was observed only at 50 dpi (**Figure 3C**). On the other hand, an IFN-γ and TNF wave was observed at 24 hpi (**Figures 3D, E**). IL-6 and IL-10 were also upregulated at 24 hpi in the 10^4^ group, and were maintained or even increased up to 50 dpi (**Figures 3F, G**). TGF-β upregulation was observed from 15 dpi to 50 dpi (**Figure 3H**).

In contrast, a slight IL-4 trigger was observed soon after the infection in the 10^6^ group (**Figure 3C**), while IL-6 and IL-10 increased around two to 10-fold (**Figures 3F, G**). In addition to TGF-β, TNF and IFN-γ, these cytokines genes exhibited high expression maintenance during the early phase time points (15 and 30 dpi) and during the chronic phase when infected with 10^6^ (**Figures 3H, E, D**). The 10^5^ group exhibited a tendency to follow the 10^6^ group pattern, although the alterations took longer to occur. A hallmark of the three experimental groups was the over-expression of all cytokine genes during the chronic phase. However, IL-4 expression intensity was much lower in the 10^4^ group in comparison to the 10^5^ and 10^6^ group (**Figure 3C**). These differences prompted us to investigate possible cytokine genes profiles associated to the disease outcome.

### IFN-γ Gene Expression and IgG Anti-*Leishmania* Influence Lesion Progression, While IL-6 Is Related to Parasite Loads

The factors underlying the clinical outcome of disease were also analyzed (**Table 1**), taking into account dependent (anti-*Leishmania* IgG, cytokines, arginase and iNOS gene expression) and independent (lesion size and skin parasite loads) variables. The applied multiple linear regression model was significant for both independent variables (p<0.05) and explained 66.7% (R²=0.667) of the total variance of lesion size and 62.6% (R²=0.626) of the parasite load. IFN-γ gene expression (coefficient β= 0.576; p=0.017) and IgG anti-*Leishmania* (coefficient β =0.375; p=0.007) were positively correlated with lesion size. IL-6 was the only cytokine influencing parasite loads (coefficient β= 0.447; p=0.028).

**Table 1:** Multivariate analysis of factors associated with skin lesion size and parasite load in hamsters infected with *Leishmania (Viannia) braziliensis*.

Variable	Lesion size (mm)			Parasite load** Eq parasites/mg skin		
	Correlation Coefficient	Standard Error	*p*	Correlation Coefficient	Standard Error	*p*
IgG anti-Leish*	**.375**	.024	**.007**	.079	.059	.591
Arginase	.343	.051	.134	-.296	.130	.234
iNOS	-.061	.008	.515	.037	.025	.740
TNF	-.093	.046	.596	-.009	.177	.961
IFN-γ	**.576**	.025	**.017**	.347	.064	.172
TGF-*β*	-.151	.054	.197	-.137	.133	.272
IL-10	-.449	.035	.068	.046	.091	.858
IL-6	.224	.039	.252	.**447**	.097	**.028**
IL-4	-.082	.022	.555	.261	.055	.086

*Anti-leishmanial IgG was evaluated by an ELISA assay; **Parasite load, enzymes and cytokines were evaluated by gene expression (qPCR) according to the Material and methods section. The correlation coefficient values were adjusted considering the inverse CT relationship. Bold values indicate the statistical signifcant results.

### Cytokine Gene Signatures Maybe Related to Lower Parasite Load but Not to Disease Progression

**Figure 5:**
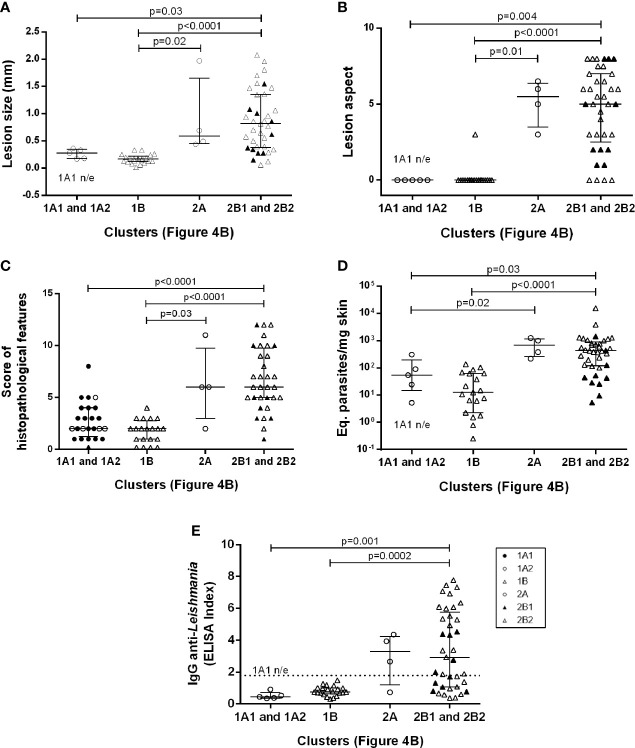
Clinical, **(A)** Lesion size, **(B)** Lesion aspect, **(C)** Score of histopathological features; parasitological **(D)** Eq. parasites/mg skin; and immunological **(E)** IgG anti-*Leishmania* aspects of *Leishmania (Viannia) braziliensis* hamster infection clustered according to skin cytokine gene expression signatures. The hierarchy average linkage clustering method with Euclidean distance measurement was used (Wishart Research Group at the University of Alberta) based on heat map analysis, as shown in **Figure 4B** (clusters: 1A1, 1A2, 1B, 2A, 2B1, and 2B2); Biomarker gene expression (*tnf, tgfβ, ifn*γ*, il10, il6, il4, arg, inos*); Each symbol represents one animal; The lines in each scatter group represent the median and interquartile range; the dotted line indicates the cut off value for IgG quantitation; ▲ represent the 2B1 subcluster and ● represent the 1A1 subcluster. *p* – statistical significance.

We used heatmaps in an attempt to group animals presenting similar skin cytokine gene expression patterns (**Figure 4A**). The animals were clustered into 1A1, 1A2, 1B, 2A, 2B1 and 2B2, according to cytokine gene patterns (**Figure 4B**). Cluster 1A1 grouped the animals at 4- and 24hpi, without any segregation related to the different inocula. The 1A2 and 1B clusters grouped most animals at 15 dpi (19 out 23), four animals at 30 dpi (three from the 10^4^ group and one from the 10^5^ group), and three 50 dpi animals from the 10^4^ group. Even considering this heterogeneity, animals in both clusters presented only discrete footpad swelling (**Figures 5A, B**) and discrete inflammatory infiltrates (**Figure 5C**). All other animals except for one presented aparasite load equal or lower than 10^2^Eq/skin/mg, including those at 30 or 50 dpi (**Figure 5D**).

The 1A1 cluster was characterized by the up-modulation of the IL-4 gene (**Figure 4B**). In contrast, animals in the 1A2 cluster exhibited upregulated TGF-β and IL-10 expressions and slightly downmodulated IL-4 expressions. Concerning 1B cluster, most animals presented TGF-β upregulation. Four animals in this cluster (one at 30 dpi and three at 50 dpi, all from 10^4^group) exhibiting low TGF-β presented high IL-10 expressions. Therefore, at least two cytokine gene profiles seem to define these clusterings. The 1A2 cytokine signature maybe associated with higher parasite loads (albeit, non-significantly) when compared to 1B (**Figure 5D**). On the other hand, greater parasite load variability was observed in the 1B group.

Cluster 2, especially 2B2, grouped most animals in the late phase of the infection (50 dpi) or infected with higher inocula (**Figure 4B**). As expected, these animals presented more severe lesions, higher IgG anti-*Leishmania* and higher parasite loads than those in cluster 1 (**Figure 5**). A considerable variability of all parameters, however, was observed. Of utmost importance, parasite load was the main distinction between subclusters 2B1 and 2B2. The 2B1 animals (black triangle) displayed significantly lower parasite loads than those in the 2B2 cluster (p=0.0005) (**Figure 5D**). The difference between these subclusters was the downregulation ofall cytokine expressions, more pronounced for IL-6 in the 2B1 cluster when compared to the 2B2 group (**Figure 4B**). It is important to note that these cytokine signatures do not define an immune response pattern associated with disease control during the latter infection phase (**Figures 5A–C**).

### An Intense Network Interaction Is Sustained in Animals Able to Control the Disease

**Figure 6:**
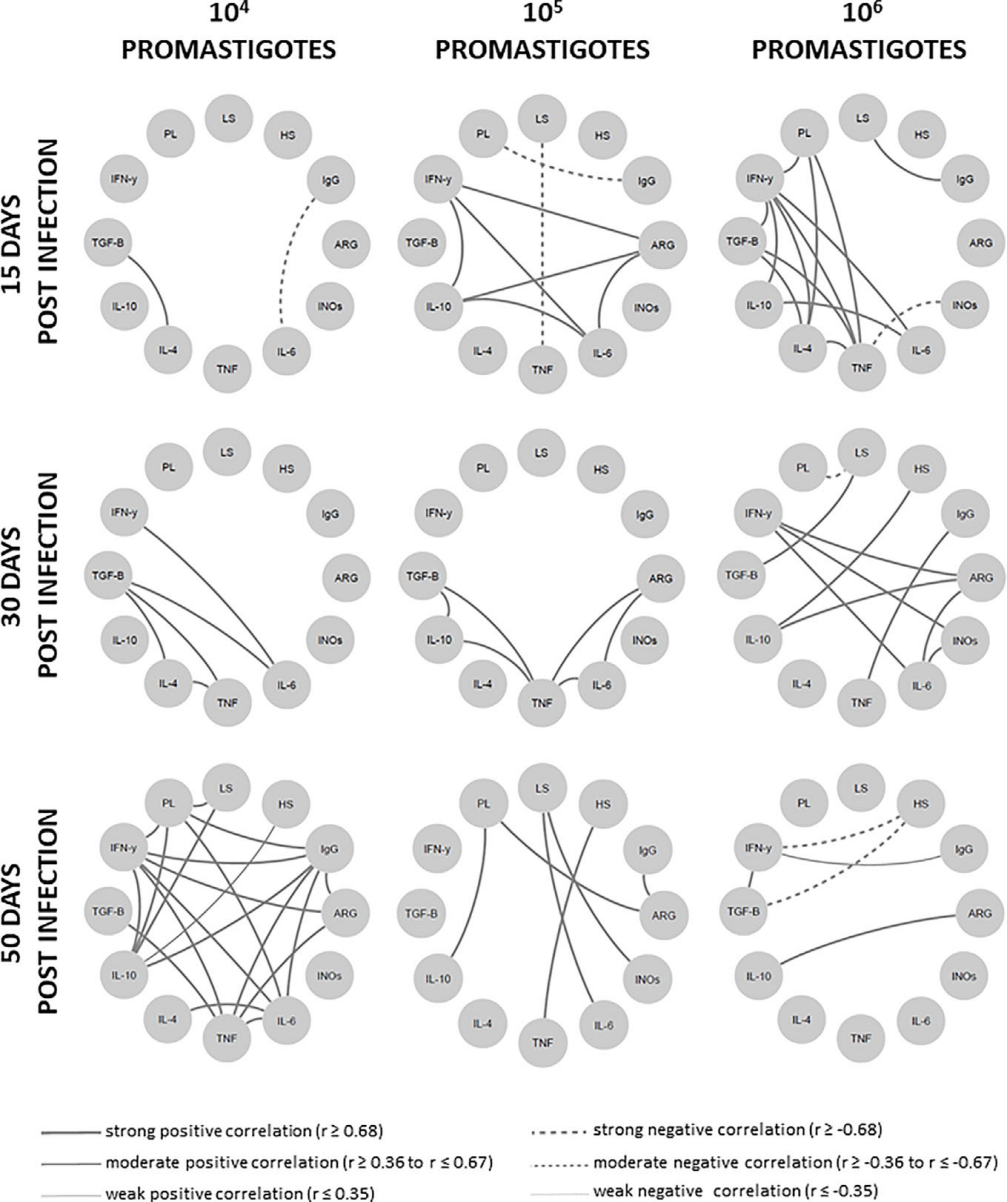
Network interaction involving clinical, parasitological and immunological biomarkers in hamsters infected with different *Leishmania Vianna braziliensis* inocula. Hamsters were evaluated during the first hours (4h, 24h), early (15 days and 30 days) and phases (50 days) post infection phases with 10^4^, 10^5^ or 10^6^ promastigotes. Significant Spearman correlations were considered when *p* ≤ 0.05, represented by lines connecting the circle nodes. The correlation index strength was represented by the connecting line thicknesses. PL, parasite load; LS, lesion size; HS, histopathology score; ARG, arginase; INOs, Oxid nitric sintase; Cytokine genes – TNF, TGF-*β*, IFN-γ, IL-10, IL-6, IL-4.

To globally evaluate the clinical immunopathological status of the disease, a network analysis of the correlation matrix including all studied biological parameters (**Figure 6**) was carried out. The 10^4^ group presented the lowest level of interactions at 15 dpi in comparison to the other groups. The number of interactions increased overtime in the 10^4^ group, contrasting to the scarce number of interactions observed in the 10^5^ and 10^6^ groups. At 50 dpi, the 10^4^ group exhibited numerous strong positive correlations involving IFN-γ and regulatory cytokines (IL-10 e TGF-β), as well as TNF/IL-6 with anti-inflammatory cytokines (IL-4). This pattern was associated with animals presenting partial parasite load (**Figure 1E**) or lesion size (**Figure 1B**) control.

**Figure 7:**
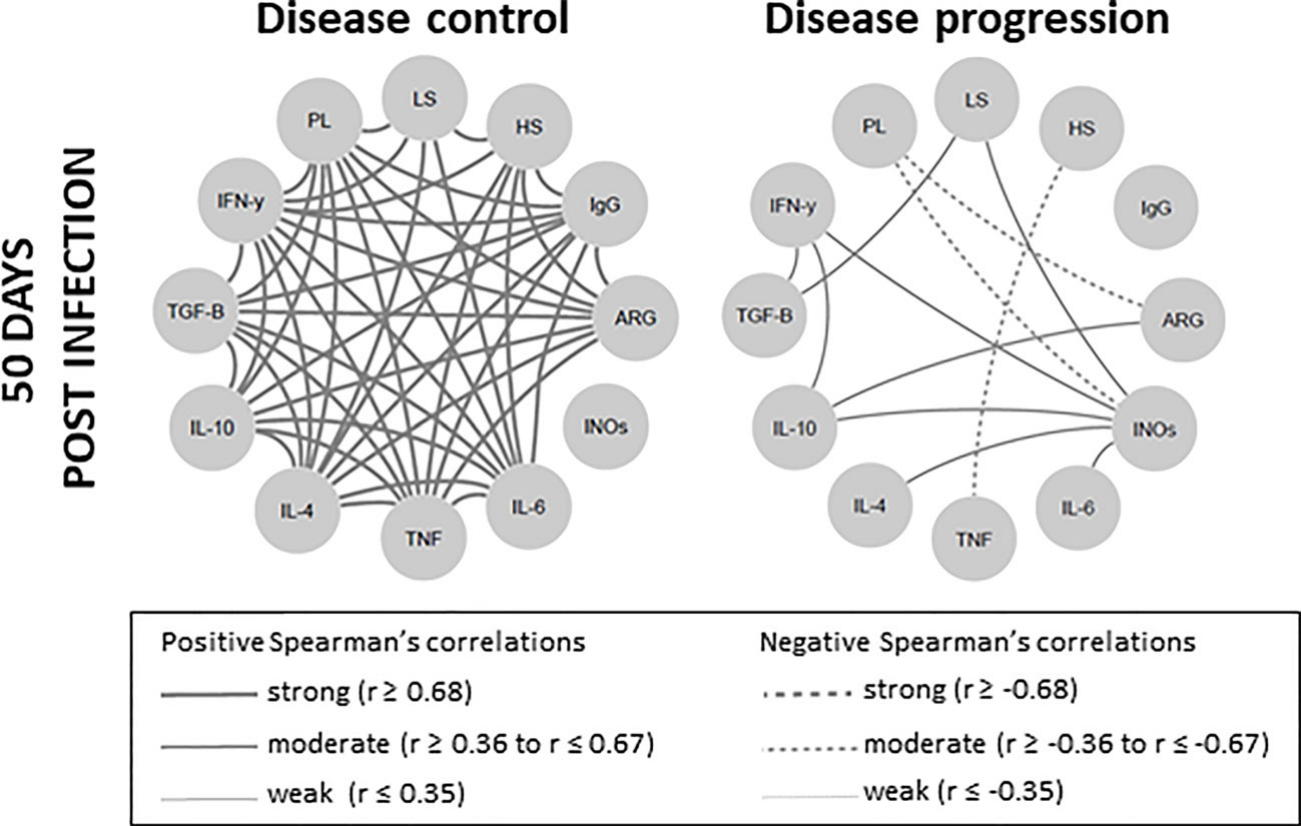
Skin lesion clinical outcome is correlated with degree of network interaction involving clinical, parasitological and immunological biomarkers in *Leishmania Viannia braziliensis*-infected hamsters. Network interactions evaluated between animals were subdivided into two clinical fates, according to lesion size and parasite load, as disease control (lesions smaller than 1mm and parasitic load <10^2^) and disease progression (lesions larger than 1mm and parasitic load > 10^2^). Significant Spearman correlations were considered when *p* ≤ 0.05 and were represented by lines connecting the circle nodes. The strength of the correlation index was represented by the connecting line thickness. PL, parasite load; LS, lesion size; HS, histopathology score; ARG, arginase; INOs, oxide nitric sintase; Cytokine genes – TNF, TGF-*β*, IFN-γ, IL-10, IL-6, IL-4.

To certify whether the disease control was associated with an intense network interaction, we distributed 50 dpi animals into two groups, namely the disease control group (lesions smaller than 1mm and parasite load <10^2^); and the disease progression group (lesions larger than 1mm and parasite load > 10^2^). The signature observed at 50 dpi inthe 10^4^ group was reproduced by the disease control group, exhibiting even more intense strong and positive interactions (**Figure 7**). On the other hand, the disease progression group presented a network displaying a lower number of moderate and positive or moderate and negative interactions, sustaining the hypothesis that immunopathogenesis is associated to a downmodulated immune response.

## Discussion

During natural *L. major* infection, it is suggested that a sand fly transfers<600 promastigotes, although higher inocula up to 100,000 have been reported (38), as observed for other *Leishmania* species (39). The infectious load can influence the severity of leishmaniasis, as higher inocula accelerate the pathogenic process (24, 31, 38), whereas lower parasite load seem to be associated with infection self-control, a common outcome in endemic areas (40). Besides, we have to consider that the site of inoculation can drive the clinical and immunological course of infection, as demonstrated in the *L. panamensis*-hamster model (26).

At the infection, the promastigotes encountered a favorable skin environment to replicate, with high arginase and TGF-β, as well as an intermediary IL-4 expression and low iNOS. However, most promastigotes were probably destroyed *in situ*, as suggested by the detection of *Leishmania* antigens scattered throughout the dermis, suggesting innate immunity action without disturbing tissue architecture. The interaction between promastigotes and the skin immune system during the very short time before their entry into host cells results in local cell stimulation. We observed that IL-10 and IL-6 genes were induced in the skin of animals infected with 10^5^ and 10^6^ inocula, while no changes were observed for TGF-β. Curiously, IL-6 positively influenced the parasite load variable. Whether IL-6 acts directly on parasite growth or *via* anti-inflammatory responses as a consequence of classical membrane receptor signaling (41) must still be clarified. PBMC from healthy humans stimulated by promastigotes also increased IL-10 production, but not IL-6, while TGF-β was down modulated (42). It is possible that the number of parasites challenging the immune system drives these phenomena, as no cytokine induction was observed in the 10^4^ group at 4 hpi. Very few amastigotes inside cells were observed at 24 hpi, as also reported elsewhere (28). On the other hand, it is important to note that only the 10^4^ group evaluated at 24 hpi presented TNF and IFN-γ gene upregulation, potentially associated to the recently transformed amastigotes. This wave could have exerted a positive influence in the next infection steps. Unfortunately, due to small size of the fragments we could not recover the live parasites from the skin lesion. These findings indicate that the infective burden influences parasite-driven cytokine activation, in which IL-4, IL-10 and IL-6 activation seems to play a role on the infection progression.

IL-4 gene induction was also different among the three infected animal groups. A slight IL-4 gene upregulation was observed at 4 and 24 hpi in the 10^5^ and 10^6^ groups, but not in the 10^4^ group. Alongside no TGF-β, IL-10 or IL-6 gene upregulation in the 10^4^ group, this points to a less permissive parasite proliferation environment. On the other hand, an intermediary basal expression of the IL-4 gene in hamster skin was confirmed (31). This cytokine has already been shown to be produced by activated skin mast cells (43, 44) or keratinocytes (45). Early IL-4 expression is noteworthy as an important resident dendritic cell activation and differentiation mediator (46, 47). Interestingly, this phenomenon was also demonstrated as a requirement for Th1 response activation (46, 48).

Although no clinical lesion signs were observed until 15 dpi, a considerable number of parasites were already detected in the lesion sites. This fits with the “silent phase” described in the CD57/BL6-*L. major* model, in which establishment of apeak load of parasites in the dermis occurred in the absence of lesion formation (19) or any overt histopathological *in situ* alterations. In humans, parasitized lymphadenopathy also precedes CL lesions (49). Herein, the parasite burden also increased through the assessed time points, indicating ongoing replication, despite high iNOS expressions, indicating no microbicidal effect (37).

After the silent phase, a TGF-β upregulation at 15 dpi was noted, when the effects of a *Leishmania*-induced T-cell response take place. At this time, the lesions became apparent and an increase in macrophage infiltrates was observed. In the *L. (V.) panamensis*-hamster model, these cells have also been reported as appearing after 10 dpi (28) to 15 dpi (50). In Balb/c macrophages, TGF-β augments the replication of *L. braziliensis* which, in turn, also produces its biologically active product (51). These authors also observed an *in vivo* exacerbation of leishmaniasis lesions, and they associated with IL-10 induction, but not IL-4, to this immune response downregulation. Corroborating this, *L. braziliensis* amastigote stimulation increased TGF-β induction 10-foldin human healthy donors, as well as IL-10, IL-6 and IL-1β mRNA, although less intensely. These authors also detected cytokine releases in these stimulated PMBC (42). It has been previously reported that six *L. braziliensis* strains induce different inflammatory and regulatory cytokines genes at 30 and 60 dpi, with lower gene induction observed for IL-6 (52). Curiously, IL-6 was the only dependent variable positively correlated with parasite load in the present study. As we utilized the same batch of a single *L. braziliensis* strain, the influence of host genetic background heterogeneity on the infection outcome can also be clearly observed.

The lowest inoculum was most frequently associated with less severe skin lesions. At 50 dpi, the lesions were small or even absentin most animals of the 10^4^ group, with similar parasite loadsin the 10^5^ and 10^6^ groups. Both the 10^5^and10^6^ groups presented similar lesions aspects, histological damage, parasite load, and anti-*Leishmania* IgG antibodies. This, however, did not occur in all animals, resulting in a very heterogeneous clinical and parasitological profile. As previously reported (31), the 10^5^ group exhibited a similar trend as the 10^6^group after the early phase. This is noteworthy, considering the high difference of the initial inocula (900.000 promastigotes), and similar behavior between the 10^5^ and 10^6^ groups was unexpected. Curiously, CL lesion parasite loads in humans do not seem to be a good predictor of disease progression (53). The mechanism driving the clinical evolution of the 10^5^ group toward to 10^6^ one seems to occur at the final early phase of the infection. The delay in disease development observed in the 10^5^ group suggests that anti-leishmanial immune mechanisms were initially triggered but were not sufficient to overwhelm the parasite burden. Other lesion analysis tools, such as changes in the proteomic spectral signature recently described for the *L. braziliensis* CL in humans, will extend our understanding of skin lesion development (54). Herein an up-regulation of almost all investigated cytokines was observed during the chronic phase. However, due to slight differences in their expressions, we considered that a systemic analysis could shed light on this phenomenon.

The heatmap clusterization according to cytokine expression intensity clearly separated animals according to disease evolution time. All animals evaluated at 4- and 24 hpi (CL1A1) and most assessed at 15 and 30 dpi (CL1A2 and 1B) were grouped. On the other hand, the CL2 group included, mainly, the animals at the end of the early and late infection phases. This cluster presented a higher cytokine expression in comparison to CL1. The exception was for IL-4, which presented much lower relative quantitation in the 10^4^ group compared to the 10^5^ and 10^6^ groups. Meanwhile, the multivariate analysis demonstrated that the IFN-γ gene, which was highly expressed, was the only independent variable that directly influences lesion size, strengthening the role of imbalanced proinflammatory responses on CL pathogenesis (55–57). Although no cytokine influenced parasite loads, CL2B1 presented lower parasite loads than CL2B2. Interestingly, IL-6 gene expression was also differentially expressed in these clusters, comparatively less expressed in CL2B1 in comparison to CL2B2. This reinforces the potential role of IL-6 and IL-4 in parasite replication in the applied hamster model. On the other hand, IL-4 induction can also be associated to a Th2 response that contributes to CL progression (30).

Cytokine inter-regulation has been demonstrated in human leishmaniasis caused by *L. braziliensis* (55). These authors observed that IL-10 and TGF-β downregulated TNF and IL-17 production, whereas IFN-γ and TNF positively induced each other. On the other hand, neutralization of IFN-γ, but not TNF, induced IL-10 release. This is in agreement with the hypothesis that a cytokine balance rather than their amounts affect the clinical outcome. The network analysis conducted herein concerning the clinical and expression of genes involved in the pathogenesis indicated that hamsters with the ability to control the *L. braziliensis* disease at 50 dpi adapted their immune response to a lower general cytokine expression coupled to a cytokine expression balance. These conditions probably maintained the regulation of cytokine effector functions through the production of relative amounts of soluble antagonistic factors. This harmonic signature involving the cytokines observed in animals that were able to control CL maybe translated by the intense network of numerous interactions based on strong and positive Spearman correlations. The absence of negative correlations also reinforces the idea of a well-modulated immune response (58–60). On the other hand, animals exhibiting disease progression displayed a reduced number of cytokine expression relationships, establishing a low immunomodulation environment. Moreover, an inverse correlation between IFN-γ gene expression and disease progression alongside an unwavering IL-4 expression throughout the evaluated timeframe, were also considered as defining an immunological hamster signature associated with CL control.

Herein we demonstrated a skin local regulation of cytokine network associated with the disease outcome in CL due to *L. braziliensis* infection. Of course, there is a need to go deep inside to elucidate the mechanism beyond this phenomena in ATL. Taken together, our findings indicate that a lower infective burden results in a cytokine profile that favors less severe cutaneous lesions in the *L. braziliensis*-hamster model, although individual susceptibility can subvert these protective mechanisms. IL-4, IL-6, IL-10, and TGF-β were associated with infection progression, while IFN-γ was correlated to tissue damage. However, more than the effect of a specific cytokine, the fine interrelation among cytokines will dictate the clinical fate of *L. braziliensis* infection. This feature should be strongly considered in vaccine designs.

## Data Availability Statement

The datasets presented in this study can be found in online repositories. The names of the repository/repositories and accession number(s) can be found in the article/**Supplementary Material**.

## Ethics Statement

The animal study was reviewed and approved by Animal Use Ethics Committee (CEUA/IOC number 032/15) - Fundação Oswaldo Cruz.

## Author Contributions

Conceived and designed the experiments: RPR-R, OCM, EFP, AMD-C, and AG-S. Performed the experiments: MPO, RPR-R, LR-V, TB-G, OCM, AFS, and LS-C. Analyzed the data: MPO, RPR-R, HGA, OCM, AMD-C, and AG-S. Wrote the paper: AMD-C, AG-S, and RPR-R. All authors contributed to the article and approved the submitted version.

## Funding

This work was supported by CNPq (Universal) - 311459/2018-8, Fundação de Amparo à Pesquisa do Estado do Rio de Janeiro: E-26/2002.944/2016 and IOC/FIOCRUZ-PAEF IOC-023-FIO-18-53.

## Conflict of Interest

The authors declare that the research was conducted in the absence of any commercial or financial relationships that could be construed as a potential conflict of interest.

## References

[1] World Health Organization. Leishmaniasis. Available at: https://www.who.int/health-topics/leishmaniasis#tab=tab_1 (Accessed in November 25th, 2020).

[2] Akhoundi M Kuhls K Cannet A Votýpka J Marty P Delaunay P. A Historical Overview of the Classification, Evolution, and Dispersion of *Leishmania* Parasites and Sandflies. PloS Negl Trop Dis (2016) 10(3):e0004349. 10.1371/journal.pntd.0004349 26937644 PMC4777430

[3] Shaw J. The Importance of Understanding Enzootic Cycles in the Epidemiology of Zoonotic Diseases With Special Reference to the American Leishmaniases. Trans R Soc Trop Med Hyg (2019) 113(3):108–9. 10.1093/trstmh/try090 30358870

[4] Romero GA Vinitius De Farias Guerra M Gomes Paes M de Oliveira Macêdo V. Comparison of Cutaneous Leishmaniasis Due to *Leishmania (Viannia) braziliensis* and *L. (V.) guyanensis* in Brazil: Clinical Findings and Diagnostic Approach. Clin Infect Dis (2001) 32(9):1304–12. 10.1086/319990 11303265

[5] Silveira FT Lainson R Corbett CE. Clinical and Immunopathological Spectrum of American Cutaneous Leishmaniasis With Special Reference to the Disease in Amazonian Brazil: A Review. Mem Inst Oswaldo Cruz (2004) 99(3):239–51. 10.1590/s0074-02762004000300001 15273794

[6] Guerra JA Prestes SR Silveira H Coelho LI Gama P Moura A. Mucosal Leishmaniasis Caused by *Leishmania (Viannia) braziliensis* and *Leishmania (Viannia) guyanensis* in the Brazilian Amazon. PloS Negl Trop Dis (2011) 5(3):e980. 10.1371/journal.pntd.0000980 21408116 PMC3050903

[7] Jirmanus L Glesby MJ Guimarães LH Lago E Rosa ME Machado PR. Epidemiological and Clinical Changes in American Tegumentary Leishmaniasis in an Area of *Leishmania (Viannia) braziliensis* Transmission Over a 20-Year Period. Am J Trop Med Hyg (2012) 86(3):426–33. 10.4269/ajtmh.2012.11-0378 PMC3284357 22403312

[8] Cupolillo E Brahim LR Toaldo CB de Oliveira-Neto MP de Brito ME Falqueto A. Genetic Polymorphism and Molecular Epidemiology of *Leishmania (Viannia) braziliensis* From Different Hosts and Geographic Areas in Brazil. J Clin Microbiol (2003) 41(7):3126–32. 10.1128/jcm.41.7.3126-3132.2003 PMC165365 12843052

[9] Rêgo FD da Rocha Lima ACVM Pereira AAS Quaresma PF Pascoal-Xavier MA Shaw JJ. Genetic Variant Strains of *Leishmania (Viannia) braziliensis* Exhibit Distinct Biological Behaviors. Parasitol Res (2018) 117(10):3157–68. 10.1007/s00436-018-6014-4 30022292

[10] Sousa R Andrade VM Bair T Ettinger NA Guimarães L Andrade L. Early Suppression of Macrophage Gene Expression by *Leishmania braziliensis* . Front Microbiol (2018) 9:2464. 10.3389/fmicb.2018.02464 30374342 PMC6196312

[11] Follador I Araújo C Bacellar O Araújo CB Carvalho LP Almeida RP. Epidemiological and Immunological Findings for the Subclinical Form of *Leishmania braziliensis* Infection. Clin Infect Dis (2002) 34(11):E54–8. 10.1086/340261 12015707

[12] Bittar RC Nogueira RS Vieira-Gonçalves R Pinho-Ribeiro V Mattos MS Oliveira-Neto MP. T-Cell Responses Associated With Resistance to *Leishmania* Infection in Individuals From Endemic Areas for *Leishmania (Viannia) braziliensis* . Mem Inst Oswaldo Cruz (2007) 102(5):625–30. 10.1590/s0074-02762007005000069 17710308

[13] Saldanha MG Queiroz A Machado PRL Carvalho LP Scott P Carvalho Filho EM. Characterization of the Histopathologic Features in Patients in the Early and Late Phases of Cutaneous Leishmaniasis. Am J Trop Med Hyg (2017) 96(3):645–52. 10.4269/ajtmh.16-0539 PMC5361539 28115669

[14] Oliveira-Neto MP Mattos MS Perez MA Da-Cruz AM Fernandes O Moreira J. American Tegumentar Leishmaniasis (ATL) in Rio De Janeiro State, Brazil: Main Clinical and Epidemiologic Characteristics. Int J Dermatol (2000) 39:506–14. 10.1046/j.1365-4362.2000.00969.x 10940114

[15] Guerra JAO Maciel MG Guerra MVF Talhari AC Prestes SR Fernandes MA. Tegumentary Leishmaniasis in the State of Amazonas: What Have We Learned and What Do We Need? Rev Soc Bras Med Trop (2015) 48:12–9. 10.1590/0037-8682-0268-2013 26061366

[16] Zajtchuket JT Casler JD Netto EM Grogl M Neafie RC Hessel CR. Mucosal Leishmaniasis in Brazil. Laryngoscope (1989) 99(9):925–39. 10.1288/00005537-198909000-00006 2671555

[17] Scott P. IFN-Gamma Modulates the Early Development of Th1 and Th2 Responses in a Murine Model of Cutaneous Leishmaniasis. J Immunol (1991) 147(9):3149–55. 1833466

[18] Launois P Ohteki T Swihart K MacDonald HR Louis JA. In Susceptible Mice, *Leishmania major* Induce Very Rapid Interleukin-4 Production by CD4^+^ T Cells Which are NK1.1^-^ . Eur J Immunol (1995) 25(12):3298–307. 10.1002/eji.1830251215 8566015

[19] Belkaid Y Mendez S Lira R Kadambi N Milon G Sacks D. A Natural Model of *Leishmania major* Infection Reveals a Prolonged “Silent” Phase of Parasite Amplification in the Skin Before the Onset of Lesion Formation and Immunity. J Immunol (2000) 165(2):969–77. 10.4049/jimmunol.165.2.969 10878373

[20] Machado P Araújo C Da Silva AT Almeida RP D’Oliveira A Bittencourt A. Failure of Early Treatment of Cutaneous Leishmaniasis in Preventing the Development of an Ulcer. Clin Infect Dis (2002) 34(12):E69–73. 10.1086/340526 12032913

[21] Bourreau E Ronet C Darcissac E Lise MC Sainte Marie D Clity E. Intralesional Regulatory T-Cell Suppressive Function During Human Acute and Chronic Cutaneous Leishmaniasis Due to *Leishmania guyanensis* . Infect Immun (2009) 77(4):1465–74. 10.1128/IAI.01398-08 PMC2663152 19168733

[22] Mendes-Aguiar CO Vieira-Gonçalves R Guimarães LH de Oliveira-Neto MP Carvalho EM Da-Cruz AM. Effector Memory CD4(+) T Cells Differentially Express Activation Associated Molecules Depending on the Duration of American Cutaneous Leishmaniasis Lesions. Clin Exp Immunol (2016) 185(2):202–9. 10.1111/cei.12798 PMC4955010 27059407

[23] Costa RS Carvalho LP Campos TM Magalhães AS Passos ST Schriefer A. Early Cutaneous Leishmaniasis Patients Infected With *Leishmania braziliensis* Express Increased Inflammatory Responses After Antimony Therapy. J Infect Dis (2018) 217(5):840–50. 10.1093/infdis/jix627 PMC5853895 29216363

[24] Bretscher PA Wei G Menon JN Bielefeldt-Ohmann H. Establishment of Stable, Cell-Mediated Immunity That Makes “Susceptible” Mice Resistant to *Leishmania major* . Science (1992) 257(5069):539–42. 10.1126/science.1636090 1636090

[25] Launois P Gumy A Himmelrich H Locksley RM Röcken M Louis JA. Rapid IL-4 Production by *Leishmania* Homolog of Mammalian RACK1-Reactive CD4(+) T Cells in Resistant Mice Treated Once With anti-IL-12 or -IFN-gamma Antibodies at the Onset of Infection With *Leishmania major* Instructs Th2 Cell Development, Resulting in Nonhealing Lesions. J Immunol (2002) 168(9):4628–35. 10.4049/jimmunol.168.9.4628 11971011

[26] Osorio Y Melby PC Pirmez C Chandrasekar B Guarín N Travi BL. The Site of Cutaneous Infection Influences the Immunological Response and Clinical Outcome of Hamsters Infected With *Leishmania panamensis* . Parasite Immunol (2003) 25(3):139–48. 10.1046/j.1365-3024.2003.00615.x 12911522

[27] Ribeiro-Gomes FL Peters NC Debrabant A Sacks DL. Efficient Capture of Infected Neutrophils by Dendritic Cells in the Skin Inhibits the Early Anti-*Leishmania* Response. PloS Pathog (2012) 8(2):e1002536. 10.1371/journal.ppat 22359507 PMC3280984

[28] Peniche AG Bonilla DL Palma GI Melby PC Travi BL Osorio EY. A Secondary Wave of Neutrophil Infiltration Causes Necrosis and Ulceration in Lesions of Experimental American Cutaneous Leishmaniasis. PloS One (2017) 12(6):e0179084. 10.1371/journal.pone.0179084 28591228 PMC5462435

[29] Launois P Maillard I Pingel S Swihart KG Xénarios I Acha-Orbea H. IL-4 Rapidly Produced by V Beta V Alpha 8 CD4^+^ T Cells Instructs Th2 Development and Susceptibility to *Leishmania major* in BALB/c Mice. Immunity (1997) 6(5):541–9. 10.1016/s1074-7613(00)80342-8 9175832

[30] Himmelrich H Launois P Maillard I Biedermann T Tacchini-Cottier F Locksley RM. In BALB/c Mice, IL-4 Production During the Initial Phase of Infection With *Leishmania major* Is Necessary and Sufficient to Instruct Th2 Cell Development Resulting in Progressive Disease. J Immunol (2000) 164(9):4819–25. 10.4049/jimmunol.164.9.481 10779790

[31] Ribeiro-Romão RP Moreira OC Osorio EY Cysne-Finkelstein L Gomes-Silva A Valverde JG. Comparative Evaluation of Lesion Development, Tissue Damage, and Cytokine Expression in Golden Hamsters (*Mesocricetus Auratus*) Infected by Inocula With Different *Leishmania (Viannia) braziliensis* Concentrations. Infect Immun (2014) 82(12):5203–13. 10.1128/IAI.02083-14 PMC4249292 25287925

[32] Gomes-Silva A Souza MA Afonso-Cardoso SR Andrade LR Dietze R Lemos E. Serological Reactivity of Different Antigenic Preparations of *Leishmania (Leishmania) amazonensis* and the *Leishmania braziliensis* Complex. Rev Soc Bras Med Trop (2008) 41(2):135–41. 10.1590/s0037-86822008000200001 18545832

[33] Ribeiro-Romão RP Saavedra AF Da-Cruz AM Pinto EF Moreira OC. Development of Real-Time PCR Assays for Evaluation of Immune Response and Parasite Load in Golden Hamster (*Mesocricetus Auratus*) Infected by *Leishmania (Viannia) braziliensis* . Parasit Vectors (2016) 9(1):361. 10.1186/s13071-016-1647-6 27350537 PMC4924296

[34] Livak KJ Schmittgen TD. Analysis of Relative Gene Expression Data Using Real-Time Quantitative PCR and the 2(–Delta DeltaC(T)) Method. Methods (2001) 25(4):402–8. 10.1006/meth.2001.1262 11846609

[35] Lopez M Inga R Cangalaya M Echevarria J Llanos-Cuentas A Orrego C. Diagnosis of *Leishmania* Using the Polymerase Chain Reaction: A Simplified Procedure for Field Work. Am J Trop Med Hyg (1993) 49(3):348–56. 10.4269/ajtmh.1993.49.348 8396860

[36] Pessenda G Silva JS. Arginase and its Mechanisms in *Leishmania* Persistence. Parasite Immunol (2020) 42(7):e12722. 10.1111/pim.12722 32294247

[37] Roma EH Macedo JP Goes GR Gonçalves JL Castro W Cisalpino D. Impact of Reactive Oxygen Species (ROS) on the Control of Parasite Loads and Inflammation in *Leishmania amazonensis* Infection. Parasit Vectors (2016) 9:193. 10.1186/s13071-016-1472-y 27056545 PMC4825088

[38] Kimblin N Peters N Debrabant A Secundino N Egen J Lawyer P. Quantification of the Infectious Dose of *Leishmania major* Transmitted to the Skin by Single Sand Flies. Proc Natl Acad Sci USA (2008) 105(29):10125–30. 10.1073/pnas.0802331105 PMC2481378 18626016

[39] Giraud E Martin O Yakob L Rogers M. Quantifying *Leishmania* Metacyclic Promastigotes From Individual Sandfly Bites Reveals the Efficiency of Vector Transmission. Commun Biol (2019) 2:84. 10.1038/s42003-019-0323-8.eCollection2019 30854476 PMC6395631

[40] Andrade-Narvaez FJ Loría-Cervera EN Sosa-Bibiano EI Van Wynsberghe NR. Asymptomatic Infection With American Cutaneous Leishmaniasis: Epidemiological and Immunological Studies. Mem Inst Oswaldo Cruz (2016) 111(10):599–604. 10.1590/0074-02760160138 27759762 PMC5066330

[41] Schaper F Rose-John S. Interleukin-6: Biology, Signaling and Strategies of Blockade. Cytokine Growth Factor Rev (2015) 26(5):475–87. 10.1016/j.cytogfr.2015.07.004 26189695

[42] Gomes CM Ávila LR Pinto SA Duarte FB Pereira LI Abrahamsohn IA. *Leishmania braziliensis* Amastigotes Stimulate Production of IL-1beta, IL-6, IL-10 and TGF-Beta by Peripheral Blood Mononuclear Cells From Nonendemic Area Healthy Residents. Parasite Immunol (2014) 36(5):225–31. 10.1111/pim.12109 24575815

[43] Oliveira MP Lima MC Calheiros AS Martins MA Antas PR De Luca PM. *Leishmania (Viannia) braziliensis*: Human Mast Cell Line Activation Induced by Logarithmic and Stationary Promastigote Derived-Lysates. Exp Parasitol (2005) 109(2):72–9. 10.1016/j.exppara.2004.11.011 15687013

[44] Descatoire M Hurrell BP Govender M Passelli K Martinez-Salazar B Hurdayal R. IL-4rα Signaling in Keratinocytes and Early IL-4 Production Are Dispensable for Generating a Curative T Helper 1 Response in *Leishmania major*-Infected C57BL/6 Mice. Front Immunol (2017) 8:1265. 10.3389/fimmu.2017.01265 29067025 PMC5641309

[45] Ehrchen JM Roebrock K Foell D Nippe N vonStebut E Weiss JM. Keratinocytes Determine Th1 Immunity During Early Experimental Leishmaniasis. PloS Pathog (2010) 6(4):e1000871. 10.1371/journal.ppat.1000871 20442861 PMC2861693

[46] Hurdayal R Nieuwenhuizen NE Revaz-Breton M Smith L Hoving JC Parihar SP. Deletion of IL-4 Receptor Alpha on Dendritic Cells Renders BALB/c Mice Hypersusceptible to *Leishmania major* Infection. PloS Pathog (2013) 9:e1003699. 10.1371/journal.ppat.1003699 24204259 PMC3812013

[47] Govender M Hurdayal R Martinez-Salazar B Gqada K Pillay S Gcanga L. Deletion of Interleukin-4 Receptor Alpha-Responsive Keratinocytes in BALB/c Mice Does Not Alter Susceptibility to Cutaneous Leishmaniasis. Infect Immun (2018) 86(12):e00710–18. 10.1128/IAI.00710-18 PMC6246911 30275010

[48] Biedermann T Zimmermann S Himmelrich H Gumy A Egeter O Sakrauski AK. IL-4 Instructs Th1 Responses and Resistance to *Leishmania major* in Susceptible BALB/c Mice. Nat Immunol (2001) 2:1054–60. 10.1038/ni725 11600887

[49] Barral A Guerreiro J Bomfim G Correia D Barral-Netto M Carvalho EM. Lymphadenopathy as the First Sign of Human Cutaneous Infection by *Leishmania braziliensis* . Am J Trop Med Hyg (1995) 53(3):256–9. 10.4269/ajtmh.1995.53.256 7573708

[50] Montoya A Yepes L Bedoya A Henao R Delgado G Vélez ID. Transforming Growth Factor Beta (TGF beta1) and Epidermal Growth Factor (EGF) as Biomarkers of *Leishmania (V) braziliensis* Infection and Early Therapeutic Response in Cutaneous Leishmaniasis: Studies in Hamsters. Front Cell Infect Microbiol (2018) 8:350. 10.3389/fcimb.2018.00350 30333964 PMC6176012

[51] Barral A Barral-Netto M Yong EC Brownell CE Twardzik DR Reed SG. Transforming Growth Factor Beta as a Virulence Mechanism for *Leishmania braziliensis* . Proc Natl Acad Sci USA (1993) 90(8):3442–6. 10.1073/pnas.90.8.3442 PMC46316 7682701

[52] Rêgo FD Fradico JRB Teixeira-Carvalho A Gontijo CMF. Molecular Variants of *Leishmania (Viannia) braziliensis* Trigger Distinct Patterns of Cytokines and Chemokines Expression in Golden Hamster. Mol Immunol (2019) 106:36–45. 10.1016/j.molimm.2018.12.013 30576950

[53] Pereira LOR Moreira RB de Oliveira MP Reis SO de Oliveira Neto MP Pirmez C. Is *Leishmania (Viannia) braziliensis* Parasite Load Associated With Disease Pathogenesis? Int J Infect Dis (2017) 57:132–7. 10.1016/j.ijid.2017.01.036 28167253

[54] Montoya A López MC Vélez ID Robledo SM. Label-Free Quantitative Proteomic Analysis Reveals Potential Biomarkers for Early Healing in Cutaneous Leishmaniasis. PeerJ (2019) 6:e6228. 10.7717/peerj.6228 30648003 PMC6330957

[55] Oliveira WN Ribeiro LE Schrieffer A Machado P Carvalho EM Bacellar O. The Role of Inflammatory and Anti-Inflammatory Cytokines in the Pathogenesis of Human Tegumentary Leishmaniasis. Cytokine (2014) 66(2):127–32. 10.1016/j.cyto.2013.12.016 PMC4047562 24485388

[56] Covre LP Martins RF Devine OP Chambers ES Vukmanovic-Stejic M Silva JA. Circulating Senescent T Cells are Linked to Systemic Inflammation and Lesion Size During Human Cutaneous Leishmaniasis. Front Immunol (2019) 9:3001. 10.3389/fimmu.2018.03001 30662437 PMC6328442

[57] Saldanha MG Pagliari C Queiroz A Machado PRL Carvalho L Scott P. Tissue Damage in Human Cutaneous Leishmaniasis: Correlations Between Inflammatory Cells and Molecule Expression. Front Cell Infect Microbiol (2020) 10:355. 10.3389/fcimb.2020.00355 32766167 PMC7381142

[58] Maucher M Kracher B Kühl M Kestler HA. Inferring Boolean Network Structure Via Correlation. Bioinformatics (2011) 27(11):1529–36. 10.1093/bioinformatics/btr166 21471013

[59] Vinhaes CL Oliveira-de-Souza D Silveira-Mattos PS Nogueira B Shi R Wei W. Changes in Inflammatory Protein and Lipid Mediator Profiles Persist After Antitubercular Treatment of Pulmonary and Extrapulmonary Tuberculosis: A Prospective Cohort Study. Cytokine (2019) 123:154759. 10.1016/j.cyto.2019.154759 31226436 PMC6739167

[60] Morgado FN de Carvalho LMV Leite-Silva J Seba AJ Pimentel MIF Fagundes A. Unbalanced Inflammatory Reaction Could Increase Tissue Destruction and Worsen Skin Infectious Diseases - A Comparative Study of Leishmaniasis and Sporotrichosis. Sci Rep (2018) 8(1):2898. 10.1038/s41598-018-21277-1 29440688 PMC5811542

